# Evaluation of Common Use Brominated Flame Retardant (BFR) Toxicity Using a Zebrafish Embryo Model

**DOI:** 10.3390/toxics4030021

**Published:** 2016-09-02

**Authors:** Crystal Y. Usenko, Erika L. Abel, Aaron Hopkins, Gerardo Martinez, Jonathan Tijerina, Molly Kudela, Nick Norris, Lana Joudeh, Erica D. Bruce

**Affiliations:** 1 Department of Biology, Baylor University, Waco, TX 76798, USA; erika_abel@baylor.edu (E.L.A.); Aaron_Hopkins@baylor.edu (A.H.); Gerardo_Martinez@baylor.edu (G.M.); makudela@utmb.edu (M.K.); Nicholas1.Norris@utsw.edu (N.N.); Ljoudeh@medicine.tamhsc.edu (L.J.); 2 Department of Environmental Science, Baylor University, Waco, TX 76798, USA; Erica_Bruce@baylor.edu; 3 School of Medicine, Stanford University, Palo Alto, CA 94305, USA; jdt2015@stanford.edu

**Keywords:** brominated flame retardant, zebrafish, oxidative stress

## Abstract

Brominated flame retardants (BFRs) are used to reduce the flammability of plastics, textiles, and electronics. BFRs vary in their chemical properties and structures, and it is expected that these differences alter their biological interactions and toxicity. Zebrafish were used as the model organism for assessing the toxicity of nine structurally-diverse BFRs. In addition to monitoring for overt toxicity, the rate of spontaneous movement, and acetylcholinesterase and glutathione-*S*-transferase (GST) activities were assessed following exposure. The toxicities of BFRs tested can be ranked by LC50 as tetrabromobisphenol A (TBBPA) < 4,4′-isopropylidenebis[2-(2,6-dibromophenoxyl)ethanol] (TBBPA-OHEE) < Pentabromochlorocyclohexane (PBCH) < 2-ethylhexyl 2,3,4,5-tetrabromobenzoate (TBB) < hexabromocyclododecane (HBCD) < hexabromobenzene (HBB) < Tetrabromophthalic anhydride (PHT4). No adverse effect was observed in di(2-ethylhexyl) tetrabromophthalate (TBPH) or dibromoneopentyl glycol (DBNPG)-treated embryos. The rate of spontaneous movement was decreased in a concentration-dependent manner following exposure to four of the nine compounds. GST activity was elevated following treatment with PBCH, TBBPA, HBCD, and HBB. The results indicate that exposure to several BFRs may activate an antioxidant response and alter behavior during early development. Some of the BFRs, such as TBBPA and TBBPA-OHEE, induced adverse effects at concentrations lower than chemicals that are currently banned. These results suggest that zebrafish are sensitive to exposure to BFRs and can be used as a comparative screening model, as well as to determine alterations in behavior following exposure and probe mechanisms of action.

## 1. Introduction

Brominated flame retardants (BFRs) are used in a wide variety of consumer products, including electronics, textiles, insulation, and carpets to raise the ignition temperature of materials [1]. There are four main types of flame retardant chemicals: inorganic salts, organophosphorus compounds, nitrogen-based compounds, and halogenated compounds. Typically, halogenated flame retardants contain chlorine and/or bromine (polychlorinated biphenyls, polybrominated diphenyl ethers, etc.). Polybrominated diphenyl ethers (PBDEs) were used for over 30 years, though most were phased out or banned worldwide in the last decade [2]. Since the mandated phase-out of PBDEs [3], the production and use of other BFRs and organophosphate flame retardants has increased [4]. Many of these flame retardants are environmentally persistent, bioaccumulative, and potentially toxic, increasing concern over possible adverse effects on human health [5,6].

Great variety in chemical structure of brominated flame retardants exists, resulting in a wide range of physicochemical properties. Since most flame retardants are not covalently bound to the products they are designed to protect, the potential for volatilization or leaching from products over time is significant. As a result, BFRs have been reported in biological samples, including breastmilk and cord blood, and house dust and sediments worldwide [7,8,9,10]. Furthermore, many of these chemicals are able to undergo long-range transport in the environment and, thus, are present in the Arctic and high-altitude lakes [2,8,11].

Of the BFRs, tetrabromobisphenol A (TBBPA) is produced in the highest volume, particularly with the ban of PBDEs and hexabromocyclododecane (HBCD) under Europe’s REACH program (Registration, Evaluation, Authorization, and Restriction of Chemicals). TBBPA and HBCD are the most commonly used BFRs in the US, though many more are used in mixtures and detected as environmental contaminants. For instance, Firemaster 550 and Firemaster BZ-54 are predominantly comprised of two chemicals: 2-ethylhexyl 2,3,4,5-tetrabromobenzoate (TBB) and di(2-ethylhexyl) tetrabromophthalate (TBPH). TBB and TBPH were detected in 50% of the air samples over the Great Lakes between 2008 and 2010 [12]. Paired with the ability to bioaccumulate and persist in the environment, these results suggest that an increase in concern over potential adverse effects of BFRs is warranted.

The purpose of the present study was to evaluate the toxicity of a variety of common-use used brominated flame retardants. PBDEs and polychlorinated biphenyls (PCBs) have been shown to disrupt hormone homeostasis and neurological development [13,14], leading to questions of the biological interactions of other brominated flame retardants. Evaluation of BFR toxicity in an in vivo model allows comparison of these preferred chemicals to banned PBDEs and other compounds of concern. In this study, we have utilized zebrafish (*Danio rerio*) during early stages of development as a model organism for comparison of adverse effects of nine selected BFRs on development and behavior. Zebrafish offer the robustness of a whole-organism model with the high-throughput capability comparable to cell culture. Further, use of zebrafish as a model organism that can be quickly and easily manipulated for toxicological and pharmacological testing, and development is rapidly expanding [15]. Finally, zebrafish have high fecundity with translucent embryos, which allows for easy identification of both malformations and developmental anomalies [16].

We hypothesized that the nine chemicals selected as a representation of BFRs with varying physicochemical properties, usage, and production would exert variable effects on embryo viability. Zebrafish embryos were exposed to the selected BFRs at varying concentrations, and mortality, developmental malformations, and behavioral alterations were assessed. In addition, we investigated glutathione-*S*-transferase (GST) and acetylcholinesterase (AChE) enzyme activities to probe potential mechanisms of action. AChE activity is a commonly used ecotoxicology assay for probing the neuro-muscular junction; GST activity was assessed as an indicator of oxidative stress.

## 2. Materials and Methods

### 2.1. Fish Care and Maintenance

Adult tropical 5D zebrafish were maintained at 28 °C on a 16:8 light:dark photoperiod in a recirculating water system. Deionized water with 0.6% sea salt was used to maintain the conductivity at 500 μS/cm and a pH of 7.0. The fish were fed *Artemia salina* twice daily. The fish were spawned in tanks, and embryos were collected daily within two hours of spawning and maintained in Petri dishes until use. All rearing and exposure protocols were approved by the Institutional Animal Care and Use Committee.

### 2.2. Chemicals and Materials Used

All BFRs used in this study were purchased from AccuStandard (New Haven, CT, USA) in neat form, dissolved in DMSO (Sigma, St. Louis, MI, USA), and stored in the dark at room temperature. The full names, abbreviations, and properties are listed in Table 1. Protease, acetylcholine iodide (AcSCh), dithiobisnitrobenzoic acid (DTNB), dithiothreitol (DTT), ethylenediaminetetraacetic acid (EDTA), glutathione, and 1-chloro-2,4,-dinitrobenzene (CDNB) were also purchased from Sigma-Aldrich.

### 2.3. BFR Exposure Method

Exposure solutions were prepared by a two-fold serial dilution in culture water and 0.5% DMSO to achieve treatment concentrations of 1.25, 2.5, 5.0, 10, and 20 ppm BFR. Control embryos were treated with a 0.5% DMSO solvent vehicle. Nominal concentrations are reported in this study. The embryos were collected at 2 h post-fertilization (hpf) and dechorionated using a protease at 6 hpf. The chorion was removed to ensure that it did not selectively inhibit uptake of BFRs. Five embryos were placed in each well of a 48-well plate containing 0.75 mL of exposure solution in each well at 6 hpf and remained in a static exposure until 168 hpf. Exposures were held in a temperature-controlled incubator at 28 °C on a 14:10 light cycle. Embryos were evaluated daily for morphological malformations, embryo viability, and mortality. For all studies, 12 replicates per concentration per chemical were used. A Motic SMZ-161 light stereoscope (Richmond, Canada) at 20 × magnification was used to view the embryos to determine development and observe physical malformations. Embryos used for enzyme activity were exposed under the same conditions until 120 hpf. At the end of the experiments, embryos were euthanized with tricaine methanesulfonate (Sigma, St. Louis, MI, USA).

### 2.4. Spontaneous Movement Evaluation

Zebrafish embryos exhibit spontaneous movement (coiling and flexing) during the 17–30 hpf developmental window [17]. At 24 hpf, the rate of spontaneous movement in treated embryos was evaluated by visually observing each well of embryos for 30 seconds and determining the average number of flexes per fish per minute for each well. A total of 12 replicates were used for each concentration of each BFR exposure.

### 2.5. Acetylcholinesterase Activity

Acetylcholinesterase activity was determined at 120 hpf following exposure to the selected BFRs. Activity was determined following a modified Ellman Protocol [18]. The concentrations of BFRs chosen were those lower than the LC_50_ values. If mortality was not observed (up to 10 ppm), the embryos were exposed to concentrations of 10 ppm. Six wells of embryos per sample were pooled, euthanized, and frozen (*N* = 4). Protein was homogenized in 100 μL of 100 mM phosphate buffered saline (PBS) with a motorized micropestle in a microcentrifuge tube. The homogenate was vortexed and centrifuged for 10 min at 13,000 × *g*. The supernatant was isolated in a clean microcentrifuge tube. AcSCh was added to extracts for a 3 mM final concentration. The PBS/DTNB solution was pipetted into 96-well plate for each sample, and 10 μL of protein extract were added to the wells. The absorbance was measured at 405 nm after 2 and 10 min of the addition of protein to the wells. AChE activity was calculated using the following equation: OD_10_−OD_2_/OD_CAL_−OD_H2O_; where OD is the optical density of the sample at 10 or 2 min. A calibrator was used to normalize between runs (OD_CAL_) and the optical density of the PBS/DTNB solution was subtracted (OD_H2O_). AChE activity was normalized to protein concentration, which was determined using a NanoDrop 1000 Spectrophotometer (Thermo Scientific, Wilmington, DE, USA).

### 2.6. GST Activity

GST activity following exposure to BFRs was assessed in whole larval lysates using CDNB as a model substrate. The method used was an adaptation of the Habig et al. protocol [19]. BFR exposure concentrations were selected based on the same criteria as the AChE assay referenced above. GST activity from exposed larvae was assessed at 120 hpf. Six wells of four embryos per sample were pooled and euthanized, and the protein extracted. Four separate sets of wells were used to total *N* = 4 biological replicates. Protein was extracted in 150 μL cold homogenization buffer (80 mM Tris, 250 mM sucrose, 0.2 mM DTT, and 1 mM EDTA) at pH 7.4 with a motorized pestle in a microcentrifuge tube. The homogenate was centrifuged for 10 min at 13,000× *g*, and the supernatant was reserved. Lysate was added to 60 mM CDNB and 15 mM GSH in 100 mM sodium phosphate buffer (pH = 6.6). GST enzyme activity was determined using a Shimadzu UV Spectrophotometer (UV-1800; Columbia, USA), and the rate of CDNG-GSH conjugate formation was monitored at 340 nm. The activity rate was normalized to protein concentration, which was determined using a NanoDrop 1000 Spectrophotometer.

### 2.7. Statistical Analysis

Fisher’s exact test was used to determine the statistical significance of differences in the rates of mortality and malformations (*p* < 0.05, *N* = 12). Rates of spontaneous movement were analyzed for statistically significant differences using one-way analysis of variance (ANOVA), with Tukey’s post-hoc test (*N* = 12). AChE and GST activities were analyzed for statistical significance using one-way ANOVA, with an *N* = 4 for each study.

## 3. Results

### 3.1. Embryo Mortality and Malformations

**Figure 1:**
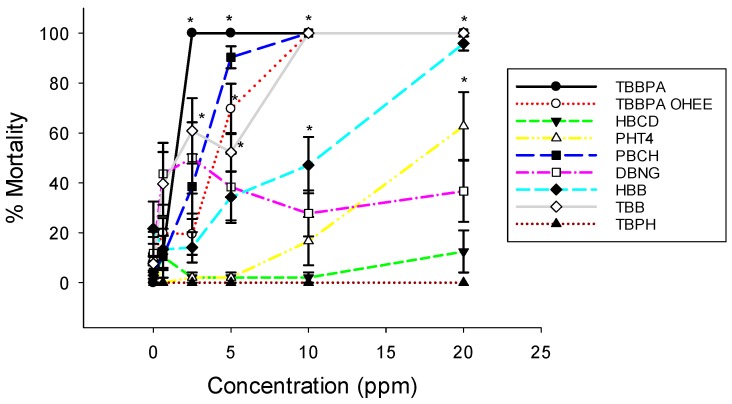
Concentration-response curves of the nine selected Brominated flame retardants (BFRs). Exposure to most BFRs resulted in a concentration-dependent increase in rate of mortality over 168 h of exposure. Exposure to TBBPA, PBCH, TBBPA-OHEE, and TBB induced the highest rate of mortality at the lowest concentration. * *p* < 0.05, *N* = 12.

Following exposure to either BFR or vehicle control, embryos were evaluated daily until 168 hpf for mortality and malformations. TBBPA induced mortality at the lowest exposure concentration tested, resulting in the lowest calculated LC50 (1.45 ppm, Figure 1, Table 2). The derivative, TBBPA-OHEE had a similar LC50 (2.2 ppm). In contrast, DBNPG and TBPH did not induce mortality at any concentration tested (up to 20 ppm). Overall, the chemicals tested can be ranked by LC50 as TBBPA < TBBPA-OHEE < PBCH < TBB < HBCD < HBB < PHT4 << TBPH, DBNPG. In comparison to our previous studies on PBDEs, the LC50 values for TBBPA, PBCH, and TBBPA-OHEE were lower than any of the PBDEs examined [20].

**Figure 2:**
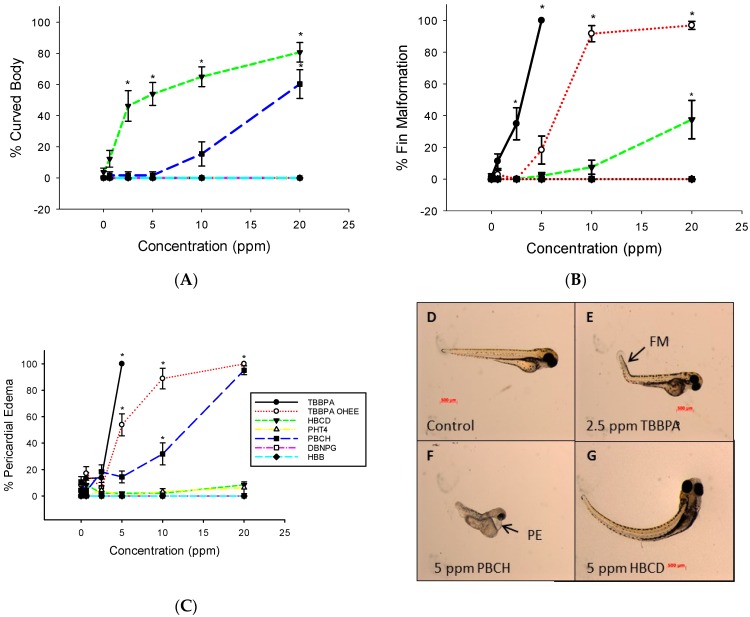
Malformations induced by BFR exposure as observed daily until 168 hpf. (**A**) HBCD and HBB induced curved body malformations; (**B**) Fin malformations were also observed in TBBPA and TBBPA-OHEE exposed embryos; (**C**) TBBPA, TBBPA-OHEE, and PBCH induced pericardial edema. Representative images of 120 hpf larvae from studies shown in panels A–C; (**D**) control, normal morphology (**E**) 2.5 ppm TBBPA, FM = fin malformation; (**F**) 5 ppm PBCH, PE = pericardial edema; and (**G**) 5 ppm HBCD, curved body malformation.

A number of the chemicals utilized in this study induced one or more malformations in the larvae. TBBPA and TBBPA OHEE exposure induced pericardial edema in embryos exposed to as little as 5 ppm concentration (Figure 2A). PBCH also induced pericardial edema, but at higher concentrations (10 and 20 ppm). TBBPA and TBPPA OHEE were the only compounds tested that induced fin malformations (2.5 and 10 ppm, respectively; Figure 2B). HBCD resulted in the highest rate of curved body malformations in a concentration-dependent manner (Figure 2C). Exposure to the other BFRs evaluated did not induce malformations, with mortality being the only physically observable effect.

### 3.2. Spontaneous Movement and Acetylcholinesterase Evaluation

**Figure 3:**
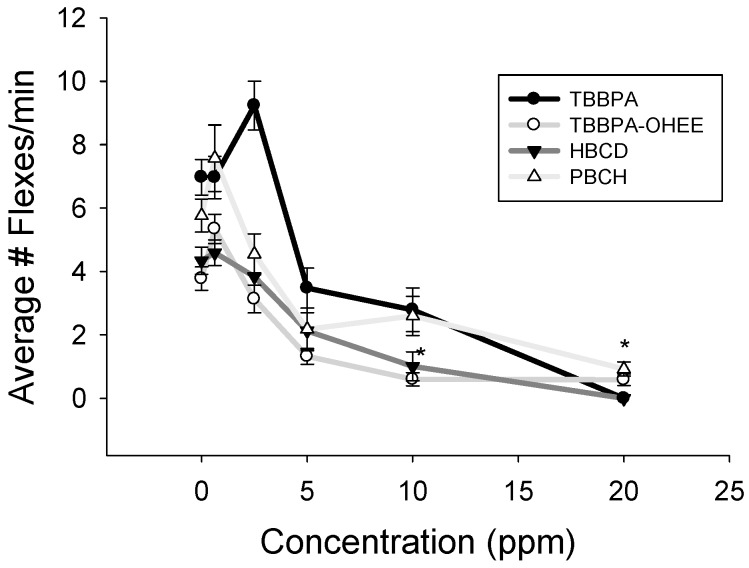
Alterations of spontaneous movement behavior at 24 hpf. Exposure to TBBPA, TBBPA-OHEE, HBCD, and PBCH decreased rates of spontaneous movement in a concentration-dependent manner. Significance is noted by * *p* < 0.05, *N* = 12. Data for chemicals that did not alter rates of spontaneous movement are not shown.

Spontaneous movement is the spontaneous flexing and coiling that zebrafish embryos exhibit from 18 hpf to 30 hpf. This behavior is also exhibited by other species during development [21]. In this study, the rate of contractions was monitored to serve as an early indicator of neurological development or stress [17]. In this study, we hypothesized that the nine BFRs would interfere to varying degrees with cell signaling required for this involuntary muscle movement. TBBPA, TBBPA OHEE, and HBCD decreased the rate of spontaneous movement in a concentration-dependent manner (Figure 3). Exposure to PBCH increased rate of spontaneous movement at 2.5 ppm, but the rate decreased at 10 and 20 ppm (Figure 3). At 10 ppm TBBPA and TBBPA-OHEE, spontaneous movement completely ceased, although the embryos were still alive at this point. Exposure to TBB, TBPH, DBNPG, PHT4, and HBB did not alter the rate of spontaneous movement at any concentration tested.

**Figure 4:**
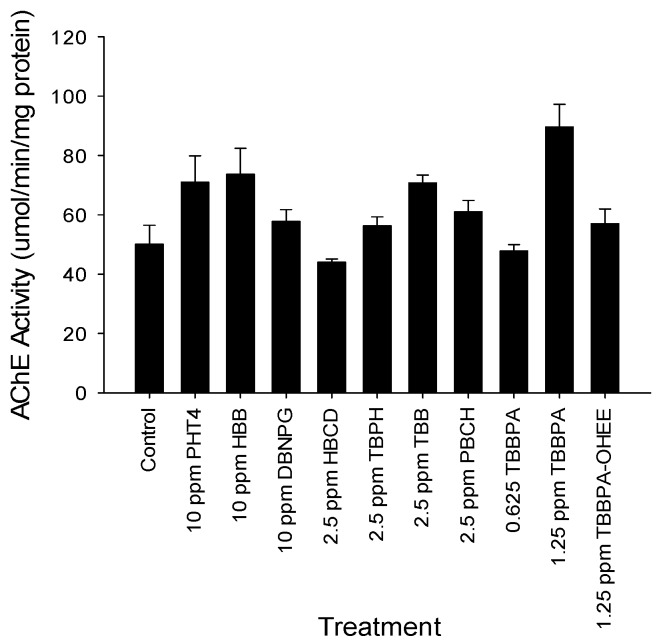
Acetylcholinesterase activity at 120 hpf. Embryos were exposed to BFRs at the indicated concentrations and AChE activity was measured at 120 hpf. Only exposure to 1.25 ppm TBBPA increased AChE activity (*N* = 4). No compounds tested suppressed AChE activity below control.

To begin to investigate the mechanistic basis for BFR effects on neuronal function, acetylcholinesterase activity was measured following exposure. Embryos were exposed to each of the nine BFRs at either 10 ppm or the lowest observable effects level in order to ensure chemical-induced mortality did not interfere with results. Acetylcholinesterase activity was not altered following exposure to any of the BFRs tested in this study (Figure 4).

### 3.3. GST Activity Evaluation

**Figure 5:**
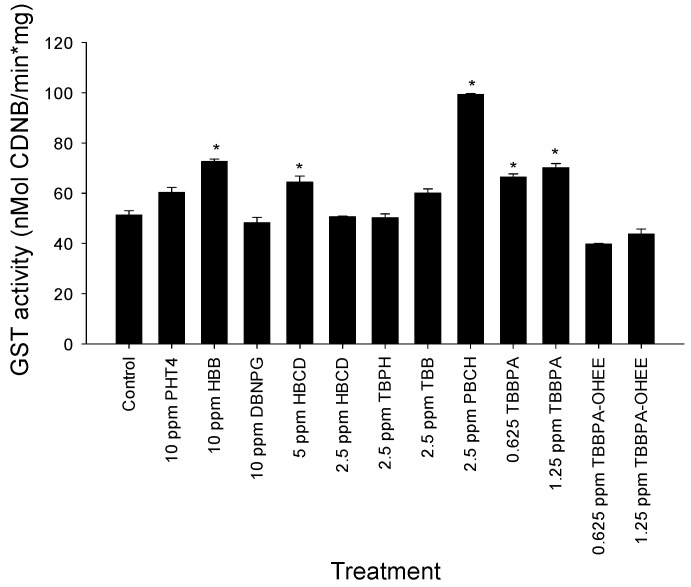
The activity of glutathione-*S*-transferase (GST) was measured at 120 hpf following exposure. Exposure to TBBPA, HBB, HBCD, and PBCH significantly increased GST activity as compared to the control. * *p* < 0.05, *N* = 4.

GST functions in the detoxification of xenobiotics and lipid peroxides. Since a number of the members of the GST enzyme family possess an antioxidant response element in the promoter region, elevation in GST expression may serve as an indicator of oxidative stress. GST activity was measured at 120 hpf in whole body lysates from larvae that had been exposed to the nine BFRs. This time point was chosen for comparison to our previous study on PBDE effects in embryonic zebrafish [20]. Exposure to 10 ppm HBB, 5 ppm HBCD, 2.5 ppm PBCH, and 0.625 and 1.25 ppm TBBPA resulted in an increased level of GST activity as compared to the control. Exposure to PBCH at 2.25 ppm resulted in the greatest elevation in GST activity when compared to the control. Although exposure to TBBPA resulted in an increase in GST activity, TBBPA-OHEE did not induce GST activity at the concentrations tested (0.625 and 1.25 ppm; Figure 5). Exposure to the remaining BFRs did not lead to statistically significant increases in GST activity when compared to the control.

## 4. Discussion

Our study demonstrates the need for further examination of the hazard posed by production and use of BFRs. The results presented here show that under a standardized treatment protocol, a high degree of variability in toxicity associated with BFR exposure exists. Further, several common-use BFRs were found to have greater overt toxicity than historical use flame retardants when examined under comparable conditions. Exposure of zebrafish embryos to BFRs led to mortality, malformations, and behavioral changes in a concentration-dependent manner. In addition, increased GST activity was also detected following exposure to select BFRs, suggesting oxidative stress as a potential mechanism of action.

Glutathione-*S*-transferase (GST) activity was evaluated in this study as an indicator of oxidative stress. Although no prior data suggest that GSTs catalyze BFR compounds, GST’s function, in general, is to catalyze the deoxification and elimination of electrophiles within the cell. As such, their expression is controlled by activation of an antioxidant responsive element (ARE) in the 5’ promoter region; therefore, elevated GST enzymatic activity often indicates induction of oxidative stress. Previous studies have shown that GST activity in zebrafish does not increase following PBDE exposure [22]. Furthermore, transcription of the pi subunit of GST (*gstpi*) was down-regulated following exposure to 6-OH-BDE47 [23]. GST activity increased in a concentration-dependent manner following exposure of scallops to TBBPA [24]; however little information is available on the alterations of GST activity for most BFRs.

TBBPA is one of the mostly commonly used flame retardants [10], yet it induced the greatest toxicity of the BFRs examined in this study. Of particular concern, the LC50 was significantly lower than that of the PBDEs as we previously reported, yet it is not yet restricted in use [20]. In our previous studies, the PBDE LC50 values ranged from 3.6 to 20 ppm (depending on the congener and number of brominations) [20]. Unlike PBDEs, TBBPA is covalently bound to the products it is used in. However, the binding efficiency leaves approximately 10% unbound within the product and, thus, able to leach out or evaporate due to its volatile nature [8]. As a result, TBBPA has been found in multiple environmental samples including sewage sludge, river water, and indoor air samples worldwide [25,26,27]. With increasing environmental concentrations, the toxicity of TBBPA, as determined in this study compared to other chemicals, is of particular concern.

Derivatives of TBBPA, such as TBBPA-OHEE, have also been found in bird, fish, and mollusk samples [10,28]. In the present study, TBBPA induced adverse effects at slightly lower concentrations than the derivatized TBBPA-OHEE; however, the types of malformations were the same and both reduced rates of spontaneous movement. One difference was noted; TBBPA induced GST activity while TBBPA-OHEE did not. These findings suggest a difference in either induction of oxidative stress by the compounds or response to oxidative stress. Other studies have demonstrated that TBBPA exposure induces oxidative stress using dichlorofluorecein diacetate and cell death (via acridine orange) in zebrafish [29,30]. Wu et al. also reported a decrease in glutathione peroxidase activity following exposure to TBBPA, which corresponds to the increase in GST activity determined in the present study [30].

In our study, HBCD exposure induced curved body malformations, in line with previous reports [31,32]. The curved body malformation was also previously observed in zebrafish following exposure to PBDEs [20]. Further, exposure to 5 ppm HBCD also resulted in a slight increase in GST activity, indicating exposure may induce oxidative stress. While further investigation is necessary, others have shown HBCD exposure increases catalase transcription, lactate dehydrogenase release (LDH), and the formation of reactive oxygen species in cell culture and zebrafish embryos [32,33]. Du et al. reported there were isomer-specific differences in toxicity of HBCD in zebrafish, with γ-HBCD being the most toxic [32].

Although it was one of the most toxic BFRs identified in the current work PBCH is, historically, the least studied. PBCH has not been extensively used as a flame retardant, nor has its toxicity been evaluated in other model systems, to our knowledge. In addition to inducing mortality in the low ppm range, exposure to 2.5 ppm PBCH induced the greatest increase in GST activity. Further characterization of PBCH-induced toxicity may be warranted if production and use increase.

HBB is a flame retardant primarily used in Japan and China and, consequently, it is not often identified in European samples [34,35]. Recently, HBB was identified in higher concentrations in the brain than in the blubber of dolphins, indicating its ability to pass through the blood brain barrier [36]. In line with our observation of HBB-induced elevation in GST activity in the present study, previous research revealed decreased superoxide dismutase and glutathione peroxidase activity in HBB exposed liver [37]. Further, in a study by Feng et al., glutathione concentration was decreased and malondialdehyde concentration was increased in a concentration-dependent manner following exposure to HBB [37]. In contrast to the other chemicals that induced GST activity (TBBPA, HBCD, and PBCH), HBB exposure did not alter spontaneous movement at any concentration tested. Given the high production volume and use of HBB in specific regions, the ability of HBB to induce oxidative stress and impact human and wildlife health should be further investigated.

In the embryonic zebrafish model, PHT4 and DBNPG did not elicit adverse effects. With a LC50 of 18 ppm, PHT4 is considerably less toxic in our model than most of the BFRs and PBDEs tested, suggesting that the human health risk associated with PHT4 exposure is comparatively low. It is heavily used as a flame retardant in urethane foam, but little to no information was previously available concerning its potential to induce toxicity [38]. DBNPG did not induce mortality or malformations in this model, and very little research has been conducted on this chemical, with most focusing on the biodegradation [39].

Exposure to the two components of the FM-550 mixture, TBPH and TBB, elicited very different responses. TBB was moderately toxic with an LC50 value comparable to most PBDEs, while TBPH did not elicit an adverse effect at any concentration tested. TBPH and TBB are often in a mixture of a 4:1 ratio by weight, and thus TBPH is found in greater abundance in the environment [40]. In fact, recently TBB and TBPH were identified as the most abundant BFRs in human hair and nails, along with BDE 99 and BDE 47 [41]. In the present study, neither chemical altered the rate of spontaneous movement or AChE activity; however, others have reported alterations in swimming behavior at 5 dpf and in the juvenile stage [31,42].

One of the benefits of using zebrafish during early development as a model organism, is the ability to evaluate alterations in behavior. Exposure to several BFRs in the current study resulted in a decrease in spontaneous movement prior to mortality. This endpoint has been demonstrated to be very sensitive and precedes mortality and the onset of malformation. In the case of the BFRs tested in this study, alterations in spontaneous movement indicated there would be a reduced rate of survival. However, further investigation into acetylcholinesterase activity as a potential target, did not result in inhibition. This assay was conducted at 120 hpf rather than 24 hpf. Noyes et al. had similar findings for TBBPA induced hypoactivity at 24 hpf, as well as hypoactivity at 120 hpf [31]. There does not appear to be a relationship between AChE activity and the alterations in behavior in the present study; however, another study found altered AChE activity following exposure to derivatives of TBBPA, indicating a neurotoxic effect of exposure [43]. It is also hypothesized that alterations in neurological function may be due to oxidative stress [44,45]. Further investigation into neurological disruption and other pathways are necessary.

## 5. Conclusions

This study compares the toxicity of nine BFRs and builds upon our previous research on PBDEs. The findings of this study suggest that the use and production of TBBPA should be reexamined, and that exposure to TBBPA may pose a risk to human and environmental health that is greater than that of PBDEs. Several of the chemicals tested, such as TBPH and DBNPG, did not induce observable adverse effects, which is encouraging given the high production rates. Future studies are needed to further assess mechanisms of action of TBBPA and HBCD. While increased GST activity is a possible indicator of oxidative stress, further testing will be required to definitively show these chemicals are causing oxidative stress in zebrafish embryos. Additionally, the mechanisms underlying alterations in early spontaneous movement should be explored. Finally, this study highlights the need for caution and comparative studies when substitute chemicals are chosen following the phase out of current-use chemicals. This concept extends to the organophosphate flame retardants and will be addressed in future studies.

## Figures and Tables

**Table 1:** The nine BFRs examined in this study and their chemical structures.

BFR ^1^	CAS	Structure
Tetrabromobisphenol A (TBBPA)	79-94-7	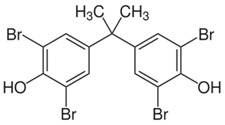
Hexabromocyclododecane (HBCD)	3194-55-6	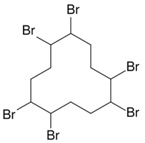
Tetrabromophthalic anhydride (PHT4)	632-79-1	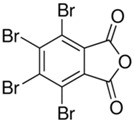
Di(2-ethylhexyl) tetrabromophthalate (TBPH)	26040-51-7	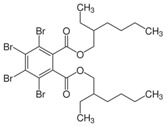
2-ethylhexyl 2,3,4,5-tetrabromobenzoate (TBB)	183658-27-7	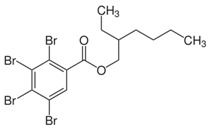
Dibromoneopentyl glycol (DBNPG)	3296-90-0	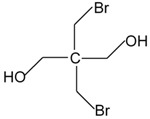
Hexabromobenzene (HBB)	87-82-1	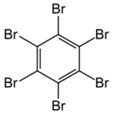
Pentabromochlorocyclohexane (PBCH)	87-84-3	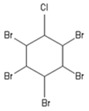
4,4′-isopropylidenebis[2-(2,6-dibromophenoxyl)ethanol] (TBBPA-OHEE)	4162-45-2	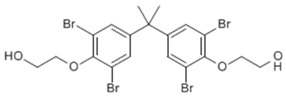

^1^ BFR, Brominated flame retardants.

**Table 2:** Summary of effects of BFR exposure in developing zebrafish. Log P is the predicted log Kow, (octanol-water partitioning coefficient).

BFR	Log P	LC50 (ppm)	EC50 (ppm)
TBBPA	7.46	1.45	0.99 (PE)
HBCD	7.47	7.7	3.35 (CB)
PHT4	4.75	18	18 (M)
TBPH	10.77	>20	>20
TBB	8.06	7.0	7.0 (M)
DBNPG	2.14	>20	>20
HBB	7.01	10.7	10.7 (M)
PBCH	4.86	2.6	2.6 (M)
TBBPA-OHEE	6.96	2.2	1.85

M = mortality; PE = pericardial edema; CB = curved body.
